# CT-based radiomics nomogram to predict proliferative hepatocellular carcinoma and explore the tumor microenvironment

**DOI:** 10.1186/s12967-024-05393-3

**Published:** 2024-09-02

**Authors:** Gongzheng Wang, Feier Ding, Kaige Chen, Zhuoshuai Liang, Pengxi Han, Linxiang Wang, Fengyun Cui, Qiang Zhu, Zhaoping Cheng, Xingzhi Chen, Chencui Huang, Hongxia Cheng, Ximing Wang, Xinya Zhao

**Affiliations:** 1 Department of Radiology, Shandong Provincial Hospital Affiliated to Shandong First Medical University, 324 Jingwuweiqi Road, Jinan, 250021 Shandong China; 2 Department of Radiology, Shandong Provincial Hospital, Shandong University, Jinan, 250021 Shandong China; 3 Department of Ultrasound, Shandong Provincial Hospital Affiliated to Shandong First Medical University, Jinan, 250021 Shandong China; 4 Department of Epidemiology and Biostatistics, School of Public Health of Jilin University, Changchun, 130021 China; 5 Department of Radiology, The First Affiliated Hospital of Shandong First Medical University & Shandong Provincial Qianfoshan Hospital, Jinan, 250014 China; 6 Department of Pathology, Shandong Provincial Hospital Affiliated to Shandong First Medical University, 324 Jingwuweiqi Road, Jinan, 250021 Shandong China; 7 Department of Gastroenterology, Shandong Provincial Hospital Affiliated to Shandong First Medical University, Jinan, 250021 Shandong China; 8 Department of Nuclear Medicine, The First Affiliated Hospital of Shandong First Medical University & Shandong Provincial Qianfoshan Hospital, Jinan, 250014 China; 9 Department of Research Collaboration, R&D Center, Beijing Deepwise & League of PHD Technology Co., Ltd, Beijing, 100080 People’s Republic of China

**Keywords:** Hepatocellular carcinoma, Radiomics, Diagnostic study, Prognostic study, Tumor microenvironment

## Abstract

**Background:**

Proliferative hepatocellular carcinomas (HCCs) is a class of aggressive tumors with poor prognosis. We aimed to construct a computed tomography (CT)-based radiomics nomogram to predict proliferative HCC, stratify clinical outcomes and explore the tumor microenvironment.

**Methods:**

Patients with pathologically diagnosed HCC following a hepatectomy were retrospectively collected from two medical centers. A CT-based radiomics nomogram incorporating radiomics model and clinicoradiological features to predict proliferative HCC was constructed using the training cohort (n = 184), and validated using an internal test cohort (n = 80) and an external test cohort (n = 89). The predictive performance of the nomogram for clinical outcomes was evaluated for HCC patients who underwent surgery (n = 201) or received transarterial chemoembolization (TACE, n = 104). RNA sequencing data and histological tissue slides from The Cancer Imaging Archive database were used to perform transcriptomics and pathomics analysis.

**Results:**

The areas under the receiver operating characteristic curve of the radiomics nomogram to predict proliferative HCC were 0.84, 0.87, and 0.85 in the training, internal test, and external test cohorts, respectively. The radiomics nomogram could stratify early recurrence-free survivals in the surgery outcome cohort (hazard ratio [HR] = 2.25; *P* < 0.001) and progression-free survivals in the TACE outcome cohort (HR = 2.21; *P* = 0.03). Transcriptomics and pathomics analysis indicated that the radiomics nomogram was associated with carbon metabolism, immune cells infiltration, *TP53* mutation, and heterogeneity of tumor cells.

**Conclusion:**

The CT-based radiomics nomogram could predict proliferative HCC, stratify clinical outcomes, and measure a pro-tumor microenvironment.

**Supplementary Information:**

The online version contains supplementary material available at 10.1186/s12967-024-05393-3.

## Introduction

Hepatocellular carcinoma (HCC) is the sixth most common neoplasms and the third leading cause of cancer-related death [1]. HCC exhibits heterogeneity at the molecular and histological levels, resulting in varying prognoses after treatment [2–4]. Recently, an integrated molecular classification was proposed to stratify HCC tumors into proliferative and nonproliferative classes [4]. Proliferative HCC is associated with *TP53* mutations, chromosomal instability, and aggressive tumor behaviors, leading to a higher recurrence rate and worse overall survival after intended curative surgery or transarterial chemoembolization (TACE) [5–7]. Conversely, nonproliferative HCC is related to the well-differentiated phenotypes and frequently shows chromosomal stability and *CTNNB1* mutations. The classification of proliferative HCC has also been beneficial for identifying the patients most likely to respond to targeted therapy or immunotherapy [4, 8]. A histological diagnosis of HCC can be attained using biopsy, but invasive procedures and random tissue sampling limit the utility of liver biopsies [9, 10]. Achieving a preoperative noninvasive diagnosis of proliferative HCC, which can guide personalized therapeutic strategies to improve clinical outcome, is an urgent and challenging issue.

Radiomics permits non-invasive characterization of tumor phenotypes by extracting high-throughput information from medical images [11, 12]. Radiomics has been applied in the diagnostics of high-risk HCC subtypes, such as microvascular invasion and vessels encapsulating tumor clusters. Radiomics can also identify the patients with shorter progression-free survival (PFS) after surgery and provide a beneficial assessment after postoperative adjuvant TACE [13–16]. However, few radiomics studies have been reported on proliferative HCC and its poor prognosis.

Radiomics has been shown to provide vast amounts of information about the tumor microenvironment and the heterogeneity of tumor cells in breast cancer [17]. Coupling the learnings from radiomics and transcriptomics could improve the interpretation of radiomics, and therefore could promote its clinical translation [18–20]. A recent study reported that magnetic resonance imaging (MRI)-based radiomics phenotypes used to predict overall survival were driven by four biological pathways in glioblastoma [21]. In addition, pathomics features extracted from histological slides could reflect the heterogeneity of tumor cells [22, 23]. To our knowledge, the relationship between radiomics and the tumor microenvironment in proliferative HCC remains unclear.

Our study aimed to construct a computed tomography (CT)-based radiomics nomogram to preoperatively predict proliferative HCC and survivals after surgery or TACE. We identified nomogram-associated genes to explore the relationship between radiomics and tumor microenvironment status. The relationship between the radiomics nomogram and the heterogeneity of tumor cells was also explored.

## Materials and methods

### Study patients

A total of 603 HCC patients from two medical centers (Shandong Provincial Hospital and Shandong Provincial Qianfoshan Hospital) and The Cancer Imaging Archive (TCIA) database were considered for inclusion in this study. The Institutional Review Board of Shandong Provincial Hospital approved this study. The requirements for informed consent were waived because of the retrospective nature of the study. The study was undertaken according to the Declaration of Helsinki.

For radiomics modeling, patients who underwent hepatectomy for HCC were retrospectively collected between January 2012 and December 2022 from two medical centers. The inclusion criteria were as follows: (a) pathological diagnosis of HCC; (b) complete preoperative contrast-enhanced abdominal CT images within 8 weeks; (c) available clinical data (detailed clinicoradiological characteristics),and (d) absence of macrovascular invasion. The exclusion criteria were as follows: (a) previous history of HCC treatment, and (b) suboptimal quality of CT images. Patients from Shandong Provincial Hospital were randomly allocated with a ratio of 7:3 into the training and internal test cohorts. And patients from Shandong Provincial Qianfoshan Hospital were used as the external test cohort. Patients with follow-up data were used as the surgery outcome cohort.

**Fig. 1:**
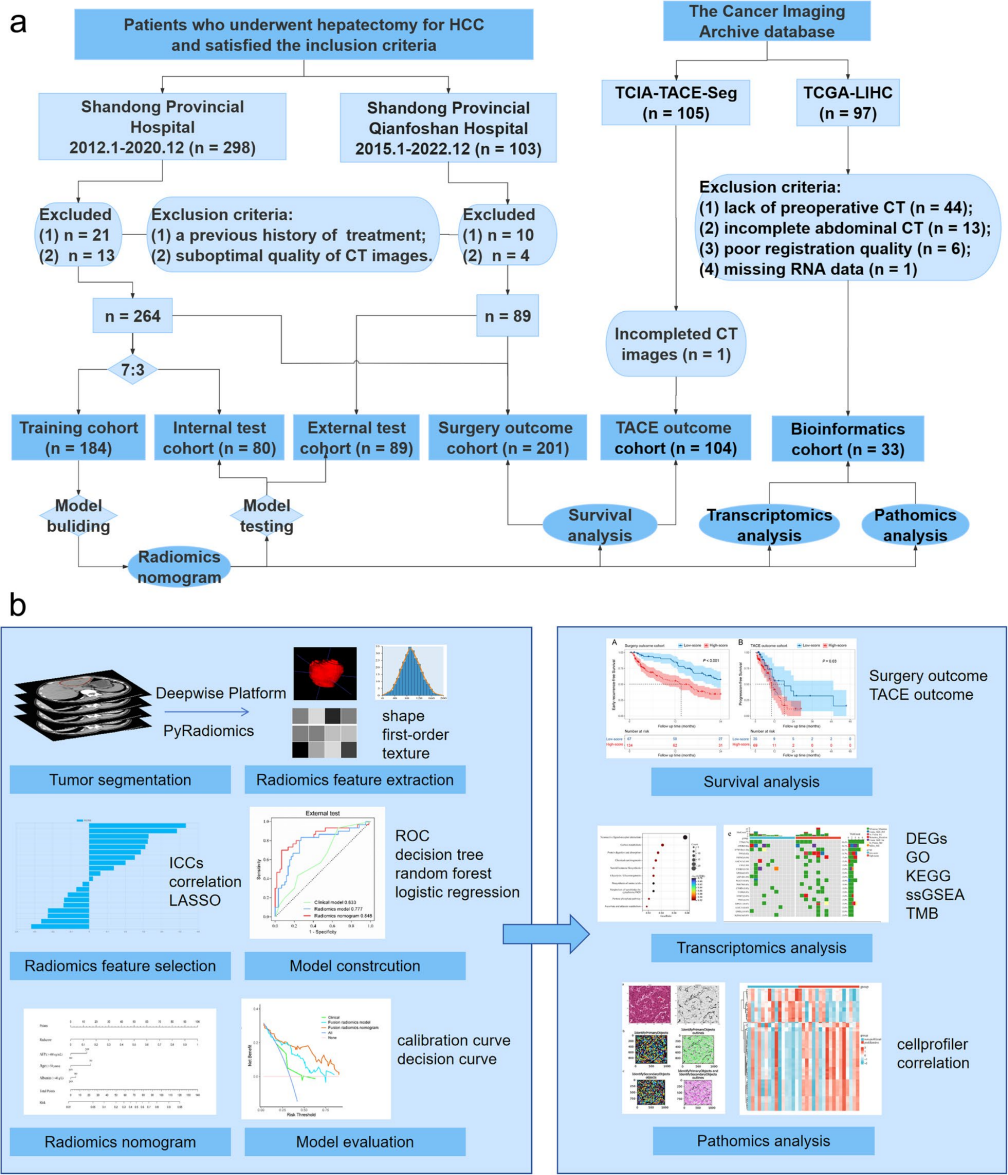
**a** Flowchart of hepatocellular carcinoma (HCC) patient enrollment. **b** The analysis workflow. Tumor lesions were segmented based on computed tomography (CT) images. Radiomics features were extracted, including first-order, shape-based, and texture features, and then selected by intraclass correlation coefficients (ICCs), correlation analysis, and least absolute shrinkage and selection operator (LASSO). Three machine learning algorithms including decision tree, random forest, and logistic regression were used to construct radiomics models, and the optimal model was combined with clinicoradiological factors to construct the radiomics nomogram. Receiver operating characteristic curve (ROC), calibration curve, and decision curve were used to evaluate the models. The radiomics nomogram was utilized for subsequent survival, transcriptomics, and pathomics analyses. Survival analyses were performed in the surgery and transarterial chemoembolization (TACE) outcome cohorts. Transcriptomics analysis included differentially expressed genes (DEGs), Gene Ontology (GO), Kyoto Encyclopedia of Genes and Genomes (KEGG), single sample gene set enrichment analysis (ssGSEA), and tumor mutation burden (TMB) analyses

Patients who received TACE therapy in the TCIA database (TCIA-TACE-Seg) were assigned as the TACE outcome cohort [24, 25]. Patients with paired CT images, RNA sequencing data and histology slides in the TCIA database and The Cancer Genome Atlas (TCGA, https://portal.gdc.cancer.gov/) database (TCGA-LIHC) were assigned as the bioinformatics cohort. The details of patient enrollment and workflow are shown in Fig. 1.

### CT image acquisition

Axial CT images including plain, arterial, portal venous, and delayed phases, were acquired from two medical centers. Patients underwent CT scanning using multidetector row CT systems (TOSHIBA Aquilion ONE, GE Discovery 750, Siemens Somatom Definition Flash, Philips Ingenuity CT, Siemens Somatom FORCE, GE Optima 620, Philips Brilliance 16). The CT scanner parameters were set as follows: tube voltage of 100 or 120 kVp, tube current of 250–400 mA, matrix of 512 × 512, rotation time of 0.25–0.60 s, and slice thickness of 1.0–5.0 mm. A non-ionic contrast agent (Omnipaque, GE Healthcare; Ultravist, Bayer Healthcare) was administered into an antecubital vein at a rate of 3.0 ml/s after obtaining the plain phase images. The arterial phase, portal venous phase, and delayed phase were acquired at 25–30, 60, and 150–180 s after injection of contrast, respectively.

### Clinicoradiological characteristic data collection

Clinical data were recorded, including age, sex, hepatitis virus infection status, presence of cirrhosis, levels of preoperative serum α-fetoprotein (AFP), aspartate aminotransferase, alanine aminotransferase, albumin, total bilirubin, and platelets, prothrombin time, and the international normalized ratio.

CT images were assessed by two experienced radiologists (X.Z. and X.W., both with more than 15 years of experience in abdominal imaging) who were blinded to histological diagnosis. CT image features including tumor diameter, capsule, margin, arterial phase hyperenhancement, peritumoral arterial enhancement, intratumor arteries, washout, intratumor necrosis, and intratumor hemorrhage were evaluated as described previously [26].

### Radiomics features extraction and model construction

**Fig. 2:**
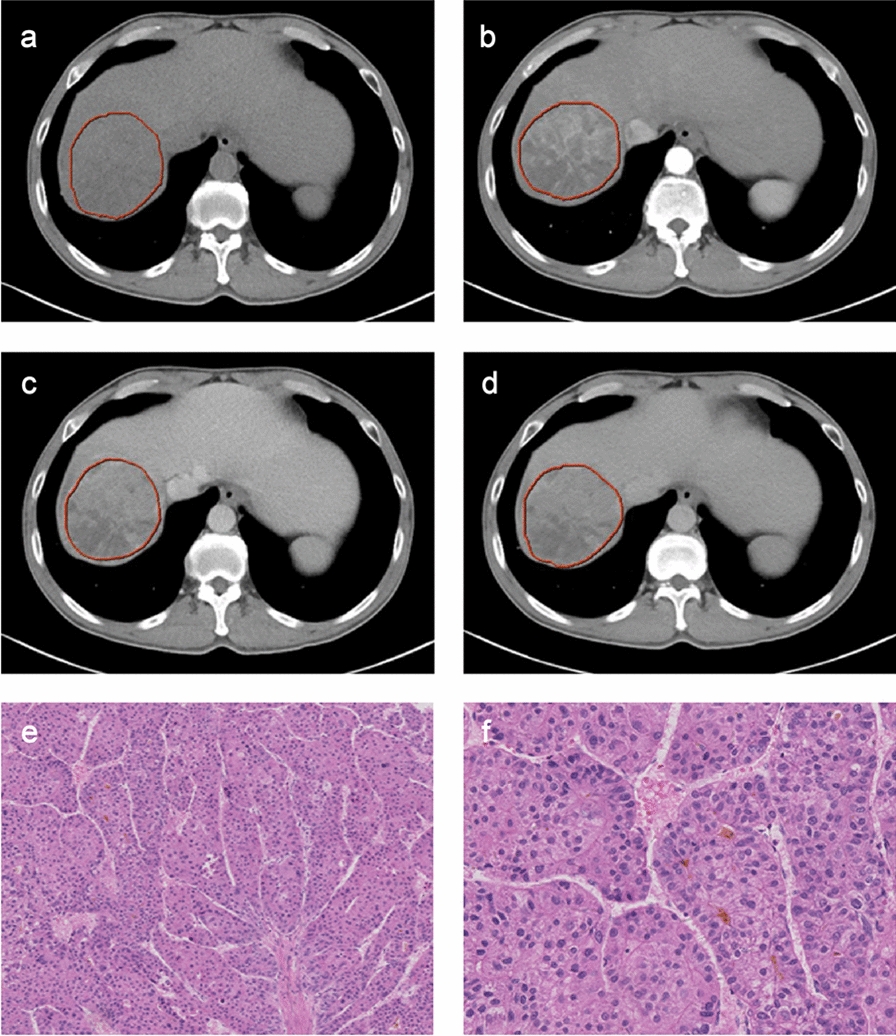
CT images and histology slides of proliferative hepatocellular carcinoma from a 45-year-old man with hepatitis B virus infection. CT images show tumor segmentations in the (**a**) plain, **b** arterial, **c** portal venous, and **d** delayed phases. Under the microscope, hematoxylin–eosin staining reveals tumor cells arranged in thick trabeculae surrounded by vascular spaces at (**e**) × 4 and (**f**) × 20 magnification. The pathological diagnosis was the macrotrabecular-massive subtype

Tumor boundaries were manually delineated on four phases of axial CT images slice by slice using the Deepwise Multimodal Research Platform (version 2.2, https://keyan.deepwise.com, Beijing Deepwise & League of PHD Technology Co., Ltd, Beijing, China; Fig. 2a–d). Two radiologists (G.W., and X.Z.) performed the segmentation in 30 randomly selected cases, and G.W. repeated the segmentation after one month later to obtain robust radiomics features with inter-observer and intra-observer intraclass correlation coefficients (ICCs) ≥ 0.8. Image preprocessing and radiomics feature extraction were performed on the Deepwise Multimodal Research Platform, which is a PyRadiomics-based software. The CT images were resampled to a voxel size of 1 × 1 × 1 mm. Radiomics features were not only extracted from original images. The first-order and texture features were extracted from nine types of filtered images, including exponential, gradient, lbp-2D, lbp-3D, log, logarithm, square, square root, and wavelet filters (Table S1). According to the imaging biomarker standardization initiative criteria, a total of 2153 radiomics features were extracted, including first-order, shape-based, and texture features (gray-level co-occurrence matrix, gray-level run-length matrix, gray-level size zone matrix, neighboring gray-tone difference matrix, and gray-level dependence matrix features). In addition, the z-score method was performed to standardize the feature values to a normal distribution.

Robust features were selected by feature correlation analysis and least absolute shrinkage and selection operator regression (LASSO) to reduce the dimensionality redundancy in the training cohort. We included the selected features to construct six radiomics models using different combination of the CT phases (Table S2). Three machine learning algorithms including decision tree, random forest, and logistic regression were used to construct models. Independent clinicoradiological characteristics (*P* < 0.05 for univariate- and multivariate logistic regression analyses) were used to build the clinical model and combined with the radiomics model to construct the radiomics nomogram.

### Histopathological evaluation

The histology slides from two medical centers were reviewed by two experienced pathologists (H.C. and F.C., with more than 10 years of experience in hepatic pathology) based on the 5th edition of the World Health Organization classification of digestive system tumors [27]. Cytokeratin 19-positive conventional HCCs were defined as conventional HCCs with more than 5% of tumor cells expressing cytokeratin 19. Macrotrabecular-massive, scirrhous, sarcomatoid, and cytokeratin 19-positive conventional HCC tumors were classified as proliferative HCC (Fig. 2e–f). Steatohepatitic, clear-cell, lymphocyte-rich, and cytokeratin 19-negative conventional HCC tumors were classified as nonproliferative HCC [5–7].

### Survival analysis

The early recurrence-free survival (RFS) in the surgery outcome cohort and PFS in the TACE outcome cohort were analyzed. Patients were stratified into low-score and high-score groups with the radiomics nomogram. The Kaplan–Meier curves were generated and log-rank tests were performed in the two outcome cohorts. Additional details are in Appendix S1.

### Exploring biological functions and pathomics features

Ninety-seven patients underwent hepatectomy for HCC in the TCIA database (TCGA-LIHC). Of these, 44 patients were excluded because of a lack of preoperative CT images, 13 because of incomplete CT phase images, six because of poor registration quality, and one because of missing mRNA data. Finally, a total of 33 patients with paired clinical data, RNA sequencing data, and CT images were included in the bioinformatics cohort. Patients were categorized into low-score (bottom 1/3 of predicted scores, n = 11) and high-score (top 1/3 of predicted scores, n = 11) groups using radiomics nomogram.

Genome-wide data of TCGA liver HCC cohort (TCGA-LIHC) were downloaded from UCSC Xena (https://xena.ucsc.edu/). The transcriptomics analysis was performed using R (version 4.2.2, http://www.rproject.org) and the Sangerbox tool [28]. The “DESeq” package was used to obtain the differentially expressed genes between the low-score and high-score groups, and a threshold of *P* < 0.05 and |log2 (fold change) |> 1 was set. We downloaded the hallmark gene set *h.all.v7.4.symbols.gmt* from the Molecular Signatures Database (http://www.gsea-msigdb.org/gsea/downloads.jsp). Functional enrichment analyses including Gene Ontology, Kyoto Encyclopedia of Genes and Genomes, and Hallmark gene set, were performed using the “clusterProfiler” package with thresholds of *P* < 0.05 and a false discovery rate < 0.25. The protein–protein interaction network was also explored in the String Database (https://string-db.org/) and the hub genes were obtained using maximal clique centrality with the Cytohuba function of the Cytoscape software [29]. We also explored the prognostic value of the hub genes using a log-rank test in TCGA. To determine the relationship between the radiomics nomogram and the tumor immune microenvironment, we used the single sample gene set enrichment analysis algorithm to obtain the infiltration levels of 28 immune cell types [30]. To further evaluate the association between the radiomics nomogram and immunotherapy in HCC, we explored the differences in the expression of immune checkpoints and *HLA* genes, and measured tumor mutation burden between the low-score and high-score groups [31–33].

The pathomics features were extracted using a pipeline in the CellProfiler software (cellprofiler.org). The detailed protocols are shown in Appendix S2.

### Statistics

Statistical analyses were performed using the SPSS (version 25.0), R (version 4.2.2, http://www.rproject.org), GraphPad (version 6.07), MedCalc (version 20.0.1), and the Deepwise Multimodal Research Platform. Categorical variables were compared using a Chi-squared test or Fisher’s exact test. The comparison of model performance was achieved using the area under the receiver operating characteristic curve (AUC), 95% confidence interval (CI), sensitivity, specificity, and continuous net reclassification improvement analyses [34, 35]. Calibration curves were performed to evaluate the fit of the models. Decision curves were used to assess the clinical utility. A *P* value less than 0.05 (two-tailed) was considered a statistically significant difference for all analyses.

## Results

### Clinical and CT radiological characteristics of patients

Of the 603 HCC patients enrolled from the two medical centers and the TCIA database, 113 patients were excluded (Fig. 1a). A total of 490 patients (median age: 58 years old [interquartile range: 49–67]; 375 men, 115 women) were finally included in our study and divided into the training cohort (n = 184), internal test cohort (n = 80), external test cohort (n = 89), TACE outcome cohort (n = 104), and bioinformatics cohort (n = 33). In addition, 201 patients from two medical centers were used as the surgery outcome cohort. The most predominant etiology was hepatic virus infection (369/490 [75.3%]). The clinical characteristics of patients is summarized in Table 1.

**Table 1:** The clinical characteristics of hepatocellular carcinoma patients

Characteristic	Training cohort (*n* = 184)	Internal test cohort (*n* = 80)	External test cohort (*n* = 89)	Surgery outcome cohort (*n* = 201)	TACE outcome cohort (*n* = 104)	Bioinformatics cohort (*n* = 33)
Age (years)	55 (47–63)	57 (48–65)	53 (48–64)	53 (46–62)	70 (59–76)	66 (56–69)
Sex (male)^a^	152 (82.6)	65 (81.3)	70 (78.7)	166 (82.6)	67 (64.4)	21 (64)
Hepatitis virus infection^a^	159 (86.4)	70 (87.5)	80 (89.9)	177 (88.1)	50 (48.1)	10 (30.3)
AFP level (ng/mL)	55.0 (3.9–1210.0)	95.1 (8.3–956.2)	18.1 (7.3–935.9)	84.8 (7.3–1210.0)	32.9 (5.9–1423.0)	19.0 (3.0–237.5)
PLT (100 × 10^9^/L)	161.5 (121.8–215.0)	157.0 (108.8–210.3)	156.0 (93.0–221.0)	162.0 (118.0–212.0)	NA	270.0 (208.5–379.5)
PT (s)	13.1 (12.3–13.9)	13.2 (12.3–14.4)	12.1 (11.3–13.3)	13.0 (12.2–14.0)	NA	8.7 (1.0–10.2)
Proliferative HCC^a^	64 (34.8)	28 (35.0)	30 (33.7)	73 (36.3)	NA	NA

Unless indicated otherwise, data are medians, with IQRs in parentheses.

*AFP* α-fetoprotein, *ALB* albumin, *NA* not available, *PLT* platelet count, *PT* prothrombin time, *TACE* transarterial chemoembolization

^a^Data are numbers of the patients, with percentages in parentheses

Histopathological evaluation revealed proliferative HCC tumors consisting of the macrotrabecular-massive (n = 73), scirrhous (n = 17), sarcomatoid (n = 1), and cytokeratin 19-positive conventional (n = 31) subtypes. Nonproliferative HCCs consisted of the steatohepatitic (n = 12), clear-cell (n = 7), lymphocyte-rich (n = 1) and cytokeratin 19-negative conventional (n = 211) subtypes. The proportions of proliferative HCCs were balanced among the training, internal test, and external test cohorts (*P* = 0.98). In the training cohort, patients with proliferative HCC were younger (*P* = 0.02) and had higher serum AFP levels (*P* = 0.003) but lower albumin levels (*P* = 0.01) compared with patients with nonproliferative HCC. Proliferative HCCs more frequently showed intratumor arteries imaging features (*P* = 0.03). There were no significant differences in other clinicoradiological characteristics between nonproliferative and proliferative HCC patients (*P* > 0.05; Table 2).

**Table 2:** Univariable and multivariable logistic regression analysis for predicting proliferative hepatocellular carcinoma in the training cohort

Characteristic	Univariable analysis	*P* Value	Multivariable analysis	*P* Value
	OR		OR	
Age (≤ 50 vs. > 50 years)	0.48 (0.25, 0.91)	0.02	0.50 (0.25, 0.99)	0.046
Sex (female vs. male)	0.52 (0.25, 1.09)	0.08		
Hepatic virus infection (absent vs present)	0.94 (0.39, 2.27)	0.89		
Cirrhosis (absent vs present)	0.98 (0.53, 1.81)	0.95		
AFP level (≤ 400 vs. > 400 ng/mL)	2.58 (1.37, 4.88)	0.003	2.00 (1.02, 3.91)	0.04
AST level (≤ 40 vs. > 40 U/L)	1.39 (0.76, 2.56)	0.29		
ALT level (≤ 50 vs. > 50 U/L)	0.88 (0.45, 1.73)	0.71		
ALB level (≤ 40 vs. > 40 g/L)	0.44 (0.24, 0.83)	0.01	0.49 (0.25, 0.94)	0.03
TB level (≤ 19 vs. > 19 μmol/L)	1.06 (0.57, 1.97)	0.85		
PLT (≤ 100 vs. > 100 × 10^9^/L)	2.29 (0.88, 5.96)	0.09		
PT (≤ 13 vs. > 13 s)	0.94 (0.51, 1.72)	0.83		
INR (≤ 1 vs. > 1)	1.44 (0.57, 3.65)	0.45		
Tumor diameter (≤ 5 vs. > 5 cm)	1.78 (0.96, 3.33)	0.07		
Tumor margin (smooth vs. non-smooth)	1.52 (0.80, 2.89)	0.20		
Intratumor necrosis (absent vs. present)	1.59 (0.82, 3.07)	0.17		
Intratumor hemorrhage (absent vs. present)	0.87 (0.37, 2.05)	0.75		
Intratumor arteries (absent vs. present)	2.04 (1.05, 3.96)	0.03	1.59 (0.79, 3.22)	0.20
Tumor capsule (absent vs. complete)	1.07 (0.52, 2.21)	0.85		
Tumor capsule (absent vs. incomplete)	0.88 (0.33, 2.32)	0.79		
Peritumoral arterial enhancement (absent vs. present)	1.45 (0.73, 2.89)	0.29		
Arterial phase hyperenhancement (absent vs. present)	0.40 (0.08, 1.90)	0.25		
Washout (absent vs. present)	0.53 (0.07, 3.82)	0.53		

Data in parentheses are 95% CIs.

*AFP* α-fetoprotein, *ALB* albumin, *ALT* alanine aminotransferase, *AST* aspartate aminotransferase, *INR* international normalized ratio, *PLT* platelet count, *PT* prothrombin time, *TB* serum total bilirubin

### Radiomics model construction to predict proliferative HCC

The selected features included in each of six radiomics models are shown in Table S2. The AUCs of radiomics models using three machine learning algorithms are shown in Table 3. Logistic regression was the best-performing classifier in fusion* (arterial and portal venous phases) and fusion (all four phases) radiomics models, and was therefore used for the subsequent analyses. The fusion radiomics model revealed AUCs of 0.77 (95% CI 0.65–0.87) and 0.78 (95% CI 0.67–0.89) in the internal and external test cohorts, respectively. The fusion radiomics model demonstrated superior performance compared to other radiomics models in the two test cohorts with a net reclassification improvement ranging from 0.51 to 0.83 (*P* < 0.05; Table 4). Radscores were derived from the radiomics models.

**Table 3:** The areas under the receiver operating characteristic curve of radiomics models to predict proliferative hepatocellular carcinoma

Model	Training cohort	Internal test cohort	External test cohort
Plain radiomics model			
Decision tree	0.68 (0.62, 0.74)	0.55 (0.45, 0.64)	0.56 (0.45, 0.66)
Logistic regression	0.67 (0.59, 0.76)	0.50 (0.36, 0.63)	0.62 (0.49, 0.75)
Random forest	1.00 (1.00, 1.00)	0.60 (0.47, 0.72)	0.63 (0.51, 0.75)
Arterial radiomics model			
Decision tree	0.76 (0.70, 0.82)	0.50 (0.38, 0.63)	0.52 (0.39, 0.64)
Logistic regression	0.70 (0.62, 0.77)	0.71 (0.60, 0.83)	0.68 (0.56, 0.81)
Random forest	1.00 (1.00, 1.00)	0.59 (0.47, 0.71)	0.59 (0.46, 0.72)
Venous radiomics model			
Decision tree	0.77 (0.70, 0.83)	0.57 (0.44, 0.69)	0.65 (0.54, 0.76)
Logistic regression	0.66 (0.58, 0.74)	0.63 (0.49, 0.76)	0.59 (0.47, 0.72)
Random forest	1.00 (1.00, 1.00)	0.55 (0.42, 0.68)	0.54 (0.40, 0.68)
Delayed radiomics model			
Decision tree	0.73 (0.65, 0.79)	0.44 (0.33, 0.57)	0.52 (0.40, 0.63)
Logistic regression	0.69 (0.60, 0.78)	0.62 (0.47, 0.75)	0.56 (0.44, 0.69)
Random forest	1.00 (1.00, 1.00)	0.56 (0.44, 0.68)	0.63 (0.51, 0.75)
Fusion* radiomics model			
Decision tree	0.79 (0.73, 0.85)	0.43 (0.31, 0.55)	0.52 (0.39, 0.64)
Logistic regression	0.71 (0.64, 0.79)	0.72 (0.59, 0.84)	0.68 (0.56, 0.81)
Random forest	1.00 (1.00, 1.00)	0.54 (0.41, 0.66)	0.59 (0.46, 0.72)
Fusion radiomics model			
Decision tree	0.84 (0.78, 0.89)	0.60 (0.49, 0.72)	0.51 (0.39, 0.64)
Logistic regression	0.82 (0.75, 0.88)	0.77 (0.65, 0.87)	0.78 (0.67, 0.89)
Random forest	1.00 (1.00, 1.00)	0.56 (0.42, 0.68)	0.67 (0.56, 0.78)

Data in parentheses are 95% CIs. Fusion* radiomics model includes features from arterial and portal venous phases. Fusion radiomics model includes features from all four phases

**Table 4:** Model comparation by continuous net reclassification improvement analysis

	Training cohort	*P* Value	Internal test cohort	*P* Value	External test cohort	*P* Value
	NRI		NRI		NRI	
Fusion radiomics model	Reference	–	Reference	–	Reference	–
Plain radiomics model	0.77 (0.49, 1.05)	< 0.001	0.83 (0.46, 1.20)	< 0.001	0.52 (0.10, 0.93)	0.01
Arterial radiomics model	0.60 (0.31, 0.89)	< 0.001	0.70 (0.27, 1.13)	0.001	0.55 (0.15, 0.96)	0.008
Venous radiomics model	0.64 (0.36, 0.93)	< 0.001	0.66 (0.23, 1.09)	0.002	0.51 (0.15, 0.88)	0.006
Delayed radiomics model	0.60 (0.31, 0.89)	< 0.001	0.76 (0.37, 1.15)	< 0.001	0.71 (0.32, 1.09)	< 0.001
Fusion* radiomics model	0.66 (0.38, 0.95)	< 0.001	0.51 (0.08, 0.94)	0.02	0.79 (0.39, 1.19)	< 0.001

Data in parentheses are 95% CIs. Fusion* radiomics model includes features from arterial and portal venous phases. Fusion radiomics model includes features from all four phases.

*NRI* net reclassification improvement.

*P* value for continuous net reclassification improvement analysis

### Radiomics nomogram building and performance testing

**Fig. 3:**
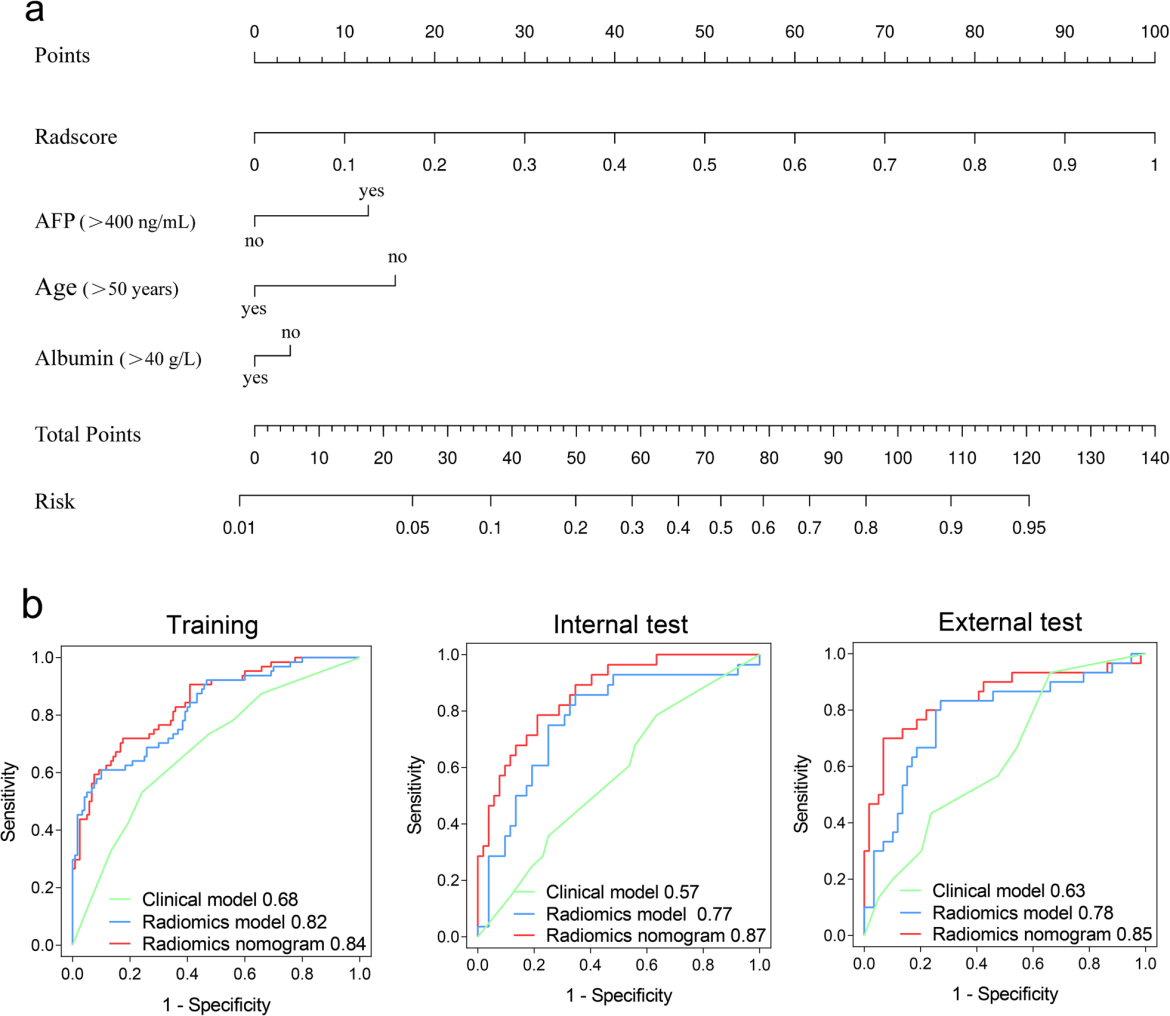
**a** Radiomics nomogram to predict proliferative hepatocellular carcinoma. **b** Receiver operating characteristic curves in the training, internal test, and external test cohorts. AFP = α-fetoprotein

Multivariate logistic regression analyses showed that age (OR = 0.50, 95% CI 0.25–0.99, *P* = 0.046), serum AFP (OR = 2.00, 95% CI 1.02–3.91, *P* = 0.04), and albumin levels (OR = 0.49, 95% CI 0.25–0.94, *P* = 0.03) were independent predictors of proliferative HCC (Table 2). The aforementioned factors were used to build the clinical model, and then combined with radscores to construct radiomics nomograms (Fig. 3a). The fusion* radiomics nomogram achieved AUCs of 0.78 and 0.76 in the two test cohorts, respectively. The fusion radiomics nomogram resulted in AUCs of 0.87 (95% CI 0.77–0.94) and 0.85 (95% CI 0.75–0.95) for the internal and external test cohorts, respectively. The AUCs of the fusion radiomics nomogram were higher than the clinical and fusion radiomics models in the two test cohorts, with net reclassification improvement ranging from 0.56 to 1.10 (*P* < 0.05; Table 5, Fig. 3b). The formulas for the models are shown in Appendix S3.

**Table 5:** Performance of clinical model, radiomics model, and radiomics nomogram to predict proliferative hepatocellular carcinoma

Model	AUC^a^	NRI	*P* value	Sensitivity (%)	Specificity (%)
Training cohort (*n* = 184)					
Clinical model	0.68 (0.60, 0.76)	0.82 (0.55, 1.10)	< 0.001	53 (34/64)	76 (91/120)
Radiomics model^b^	0.82 (0.75, 0.88)	0.18 (−0.12, 0.48)	0.24	61 (39/64)	90 (108/120)
Radiomics nomogram^c^	0.84 (0.78, 0.90)	Reference	–	72 (46/64)	83 (99/120)
Internal test cohort (*n* = 80)					
Clinical model	0.57 (0.45, 0.70)	1.10 (0.74, 1.46)	< 0.001	36 (10/28)	75 (39/52)
Radiomics model^a^	0.77 (0.65, 0.87)	0.56 (0.12, 1.00)	0.01	54 (15/28)	81 (42/52)
Radiomics nomogram^b^	0.87 (0.77, 0.94)	Reference	–	71 (20/28)	81 (42/52)
External test cohort (*n* = 89)					
Clinical model	0.63 (0.52, 0.75)	0.99 (0.61, 1.38)	< 0.001	43 (13/30)	76 (45/59)
Radiomics model^a^	0.78 (0.67, 0.89)	0.79 (0.41, 1.17)	< 0.001	53 (16/30)	85 (50/59)
Radiomics nomogram^b^	0.85 (0.75, 0.95)	Reference	–	70 (21/30)	90 (53/59)

Unless otherwise specified, data in parentheses are numbers of patients

*AUC* area under the receiver operating characteristic curve, *NRI* net reclassification improvement

*P* value for continuous net reclassification improvement analysis

^a^Data in parentheses are 95% CIs

^b^Radiomics model is based on all four phases of CT images.

^c^Radiomics nomogram includes age, serum alpha-fetoprotein level, serum albumin level, and radiomics model.

The calibration curves indicate that the fusion radiomics nomogram is well-calibrated in the two test cohorts (Fig. S1a). Decision curves suggest that the fusion radiomics nomogram resulted in enhanced clinical utility compared to the clinical and fusion radiomics models (Fig. S1b).

### Predictive value of the radiomics nomogram for survival analysis in the outcome cohorts

**Fig. 4:**
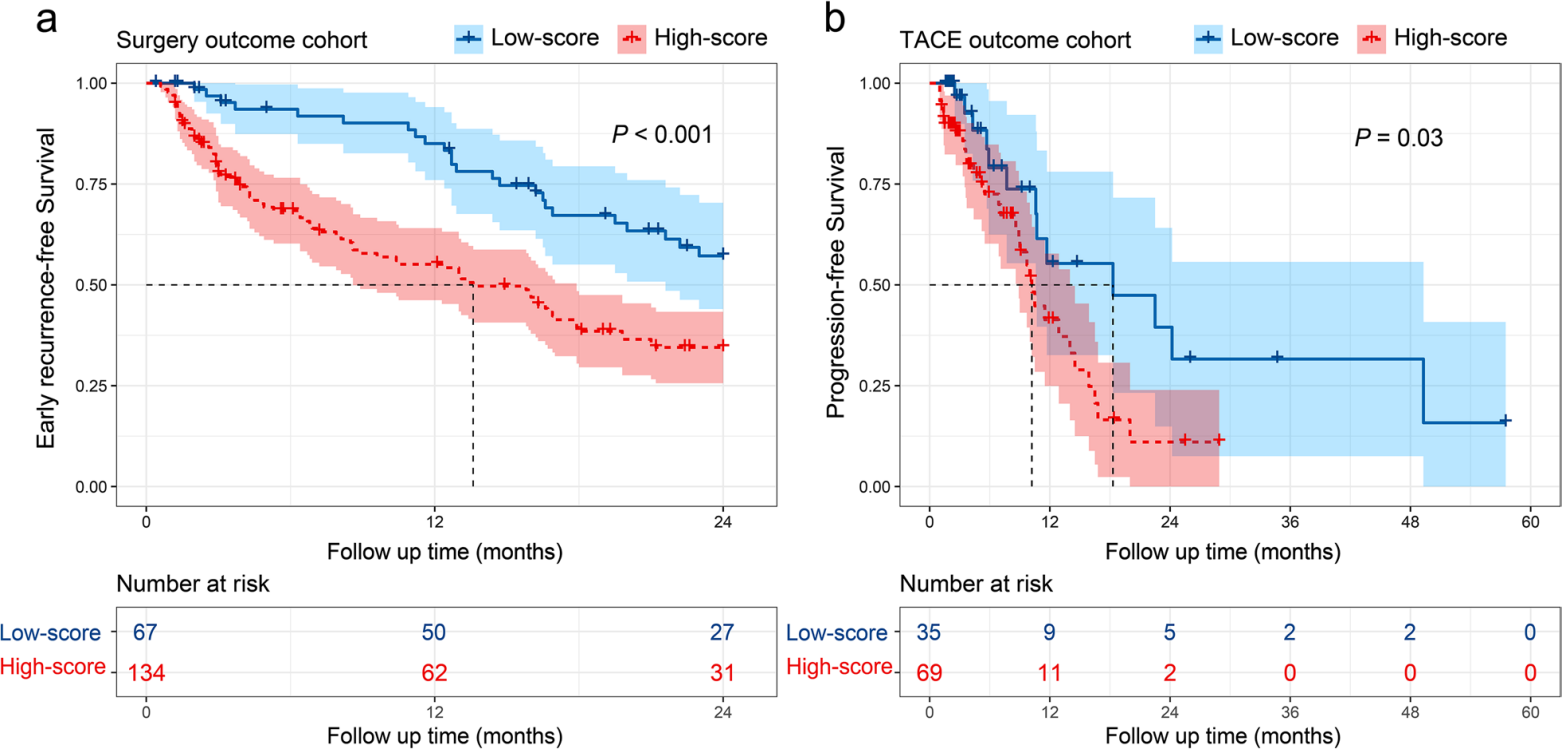
Kaplan–Meier curves of (**a**) early recurrence-free survival in the surgery outcome cohort and (**b**) progression-free survival in the transarterial chemoembolization (TACE) outcome cohort. The early recurrence-free survival and progression-free survival were estimated using scores derived from the radiomics nomogram

In the surgery outcome cohort, 201 patients completed follow-up. The median early RFS time was 17.9 months (95% CI 15.9–23.0 months) and 101 patients experienced early recurrence. In the TACE outcome cohort, 104 patients completed follow-up. The median PFS time was 11.5 months (95% CI 9.7–15.9 months) and 43 experienced tumor progression. The radiomics nomogram could stratify early RFS in the surgery outcome cohort (hazard ratio [HR], 2.25, 95% CI 1.42–3.56; *P* < 0.001) and PFS in the TACE outcome cohort (HR, 2.21, 95% CI 1.10–4.44; *P* = 0.03; Fig. 4).

### Biological functions associated with the radiomics nomogram

**Fig. 5:**
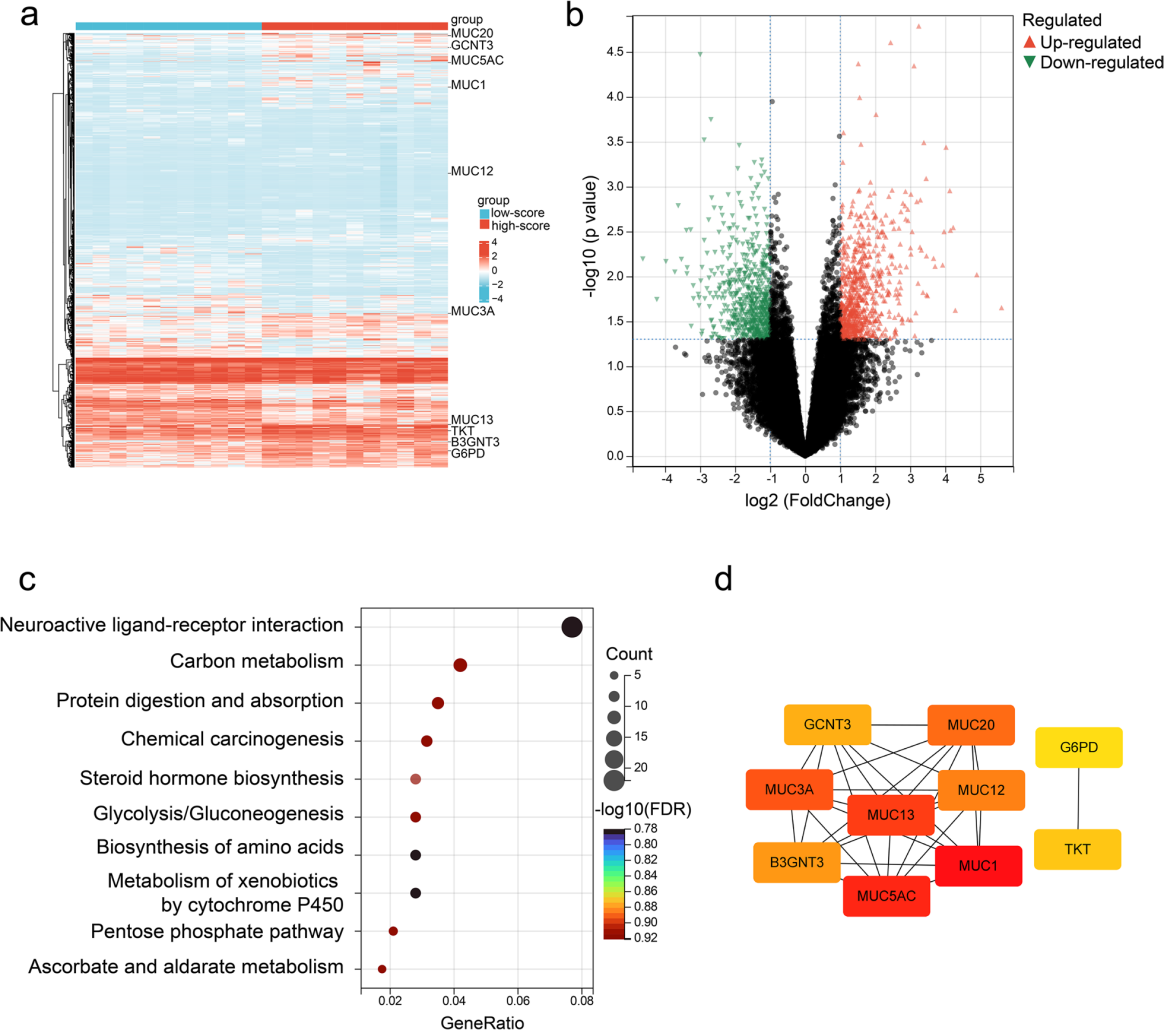
Transcriptomics analysis of biological functions associated with the radiomics nomogram in the bioinformatics cohort. **a** Heatmap and (**b**) volcano plot showing the expression levels of differentially expressed genes (DEGs) between the low-score and high-score groups. The “DESeq” package was used and thresholds of *P* < 0.05 and |log2 (fold change) |> 1 were set. **c** Functional enrichment analysis of DEGs was performed using Kyoto Encyclopedia of Genes and Genomes (KEGG). GeneRatio is the ratio of genes in this pathway to all genes. KEGG analysis was performed using the “clusterProfiler” package with thresholds of *P* < 0.05 and a false discovery rate < 0.25. **d** A protein–protein interaction network showed the top 10 hub genes identified with Cytoscape

In the bioinformatics cohort, 1367 differentially expressed genes between the low-score and high-score groups derived from the radiomics nomogram were identified (Fig. 5a–b). A functional enrichment analysis showed differentially expressed genes mainly clustered within carbon metabolism, glycolysis, oxidoreductase activity, and transmembrane transport (Fig. 5c, S2). A protein–protein interaction network analysis identified hub genes, including *MUC13*, *MUC3A*, *MUC1*, *MUC5AC*, and *G6PD* (Fig. 5d). The hub genes were mainly enriched in the glucose 6-phosphate metabolic process, carbon metabolism, and glycolysis pathways, and correlated with progression-free intervals (Fig. S3).

**Fig. 6:**
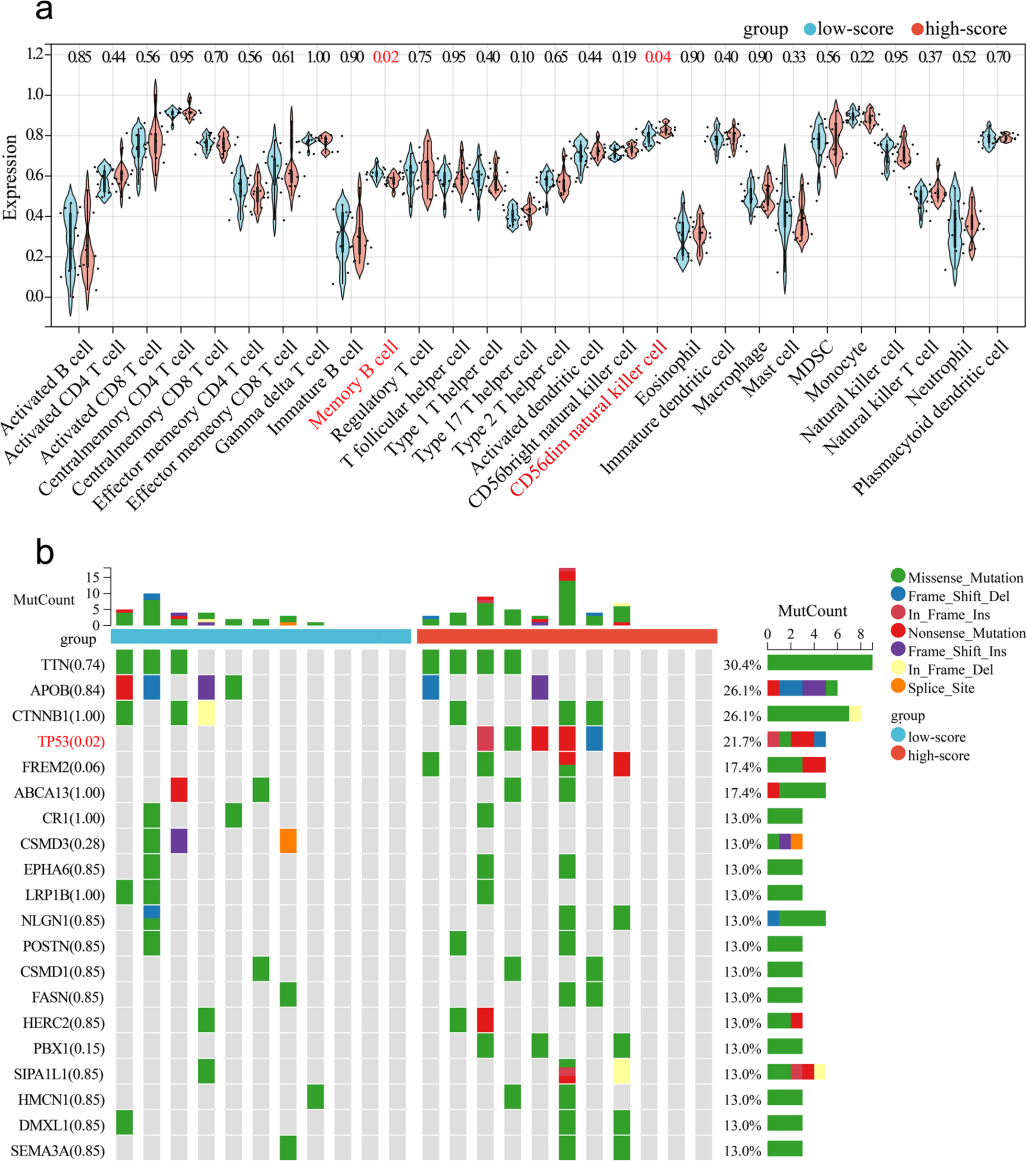
**a** Analysis of infiltrating immune cells between the low-score and high-score groups using the single sample gene set enrichment analysis algorithm. **b** Analysis of tumor mutation burden

The infiltration levels of 28 immune cell types were evaluated in the tumor samples. The lower level of memory B cells and higher levels of CD56dim natural killer cells were found in the high-score group (*P* = 0.02 and *P* = 0.04, respectively; Fig. 6a). The expression levels of the immune checkpoint genes *TNFSF9* and *HLA-C* were higher in the high-score group (*P* = 0.02 and *P* = 0.045, respectively; Fig. S4). Tumor mutation burden analysis was used to reveal somatic mutations in the tumor samples. The findings showed that *TP53* mutations were more frequent in the high-score group (*P* = 0.02; Fig. 6b).

### Identification of representative pathomics features with the radiomics nomogram

**Fig. 7:**
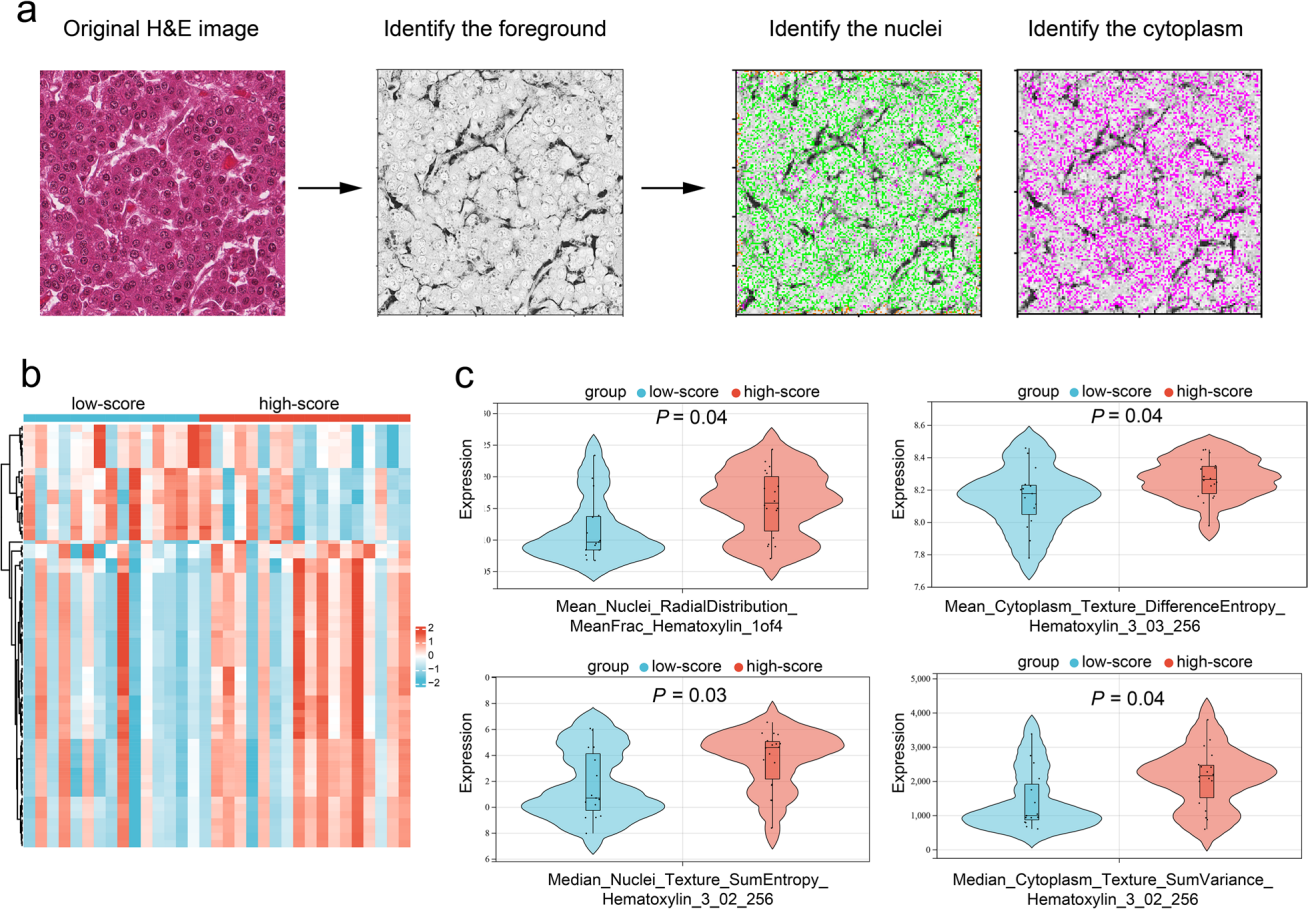
Pathomics analysis in the bioinformatics cohort. **a** The extraction procedure of the pathomics features. The foreground of targeted tissue was identified from the original hematoxylin–eosin images using CellProfiler software. Subsequently, the nuclei and cytoplasm of tumor cells were identified using the Otsu method. **b** A heatmap shows the different pathomics features between the low-score and high-score groups categorized by the radiomics nomogram. **c** Four representative pathomics features are presented

We built an automated segmentation pipeline using the Otsu method to recognize the nuclei and cytoplasm of tumor cells (Fig. 7a). A total of 1362 objective pathomics features of the recognized nuclei and cytoplasm were extracted from each histological image. A list of pathomics feature categories are shown in Table S3.

A total of 117 pathomics features were identified between the low-score and high-score groups categorized by the radiomics nomogram (Fig. 7b). Representative features included texture (SumEntropy and DifferenceEntropy), shape (BoundingBoxMaximum), radial distribution of pixel intensity (MeanFrac), and pixel intensity (MeanIntensityEdge) of the nuclei, as well as texture (DifferenceEntropy, SumVariance, Contrast, Variance, and Entropy) and pixel intensity (IntegratedIntensityEdge) of the cytoplasm (*P* < 0.05; Table S4, Fig. 7c).

## Discussion

In this study, a CT-based radiomics nomogram was constructed to predict proliferative HCC. Using this model, the AUCs were 0.84, 0.87, and 0.85 in the training, internal test, and external test cohorts, respectively. The radiomics nomogram could stratify early RFS in the surgery outcome cohort and PFS in the TACE outcome cohort. Moreover, by integrating transcriptomics and pathomics data, the radiomics nomogram was associated with carbon metabolism, immune cells infiltration, *TP53* mutations, and heterogeneity of tumor cells.

In the current clinical settings, CT and MRI imaging are preferred HCC diagnostic methods compared to biopsies [36–38]. It is difficult to identify the aggressive proliferative class without histological examinations. This is the first study to construct a CT-based radiomics models to preoperatively predict proliferative HCC. In our study, the fusion radiomics model using all four phases of CT images was built and outperformed other single-phase models (*P* < 0.05). This high performance could be explained by the fact that the complete dynamic enhancement phases are necessary for diagnosing HCC [36, 37]. In previous studies, serum AFP levels have been demonstrated to be an independent predictor of proliferative HCC, consistent with our findings [5, 6]. To assess the incremental value of radiomics over the clinical features for proliferative HCC prediction, age, AFP, and albumin were incorporated into the clinical model, and then further combined with the fusion radiomics model to establish a radiomics nomogram. The radiomics nomogram showed a superior predictive capability compared to the clinical model with higher AUCs (internal test: 0.87 vs. 0.57, *P* < 0.001; external test: 0.85 vs. 0.63, *P* < 0.001) and a higher clinical net benefit. The nomogram also achieved higher AUCs than two previously reported clinical models (0.77 and 0.80, respectively) [6, 39]. Two recent studies found that radiomics nomogram can predict microvascular invasion more accurately than the clinical model [40, 41]. These findings indicate that CT-based radiomics can add incremental value to the clinical features in predicting the histological subtypes in HCC patients. Additional validation in diverse patient populations is necessary to establish the nomogram’s robustness. Collaborations with multiple institutions or utilization of larger databases to enhance the external validation process and strengthen the nomogram’s reliability across diverse patient cohorts can be conducted.

A previous study reported that proliferative HCC is associated with intrahepatic recurrence and extrahepatic metastasis after surgery [5]. In our study, patients were divided into high-score and low-score groups based on a radiomics nomogram. HCC patients in the high-score group had shorter early RFS and PFS than those in the low-score group in the surgery and TACE outcome cohorts, respectively (*P* < 0.001 and *P* = 0.03, respectively). This suggests that the high-score patients are not eligible for hepatectomy or TACE alone, and other alternative treatments should be considered, such as postoperative adjuvant therapies, liver transplantation, and a combination of sorafenib with TACE [42–45]. Zhang et al. found that the high-risk patients identified by a radiomics nomogram could benefit from postoperative adjuvant TACE to decrease early recurrence [16]. Our study primarily focused on prediction and stratification, and further investigation into the nomogram’s direct clinical utility, such as its impact on treatment decisions or patient outcomes, would be valuable. Prospective studies could be conducted to assess how the nomogram’s predictions influence treatment strategies, patient monitoring, and overall clinical management. It is worth mentioning that the TACE outcome cohort was obtained from American patients, where the main etiology of HCC is typically not from hepatitis B virus infection.

Increasing evidence indicates that radiomics features extracted from tumors are related to underlying biological functions [17, 21]. Our study revealed that radiomics nomogram-associated differentially expressed genes are involved in carbon metabolism and glycolysis. This suggests that proliferative HCC may be aggressive if sufficient energy from glycolysis is achieved, which is the main way that tumor cells produce ATP from glucose [46, 47]. In terms of the tumor immune microenvironment, immune cell infiltration plays a crucial role in tumor initiation and progression [48]. HCC within the context of a compromised immune microenvironment is more likely to demonstrate intrahepatic recurrence and extrahepatic metastasis, causing cancer-related symptoms such as pain from bone metastases) [44, 48]. Low tumoral density of B cells in cancer patients is associated with poor clinical outcomes [49]. Recent studies found that programmed cell death protein-1, an exhaustion marker of T cells, was also highly expressed on tumor-infiltrating natural killer cells in digestive tract cancers [50, 51]. In our study, a low prevalence of memory B cells and more CD56dim natural killer cells were found in the high-score group, which contributed to the formation of the immune suppressive microenvironment. Feng et al. found that the macrotrabecular-massive subtype prediction radiomics model was associated with humoral immune dysregulation, including reduced immunoglobulin synthesis and decreased B-cell infiltration [52]. Xia et al. also reported that microvascular invasion development may be associated with decreased immune cells in the tumor microenvironment [40]. In future work, clinical features and an immune-comprised status should be integrated with radiomics features to predict treatment responses and clinical outcomes in HCC patients.

Immune therapy has demonstrated encouraging response rate, durability, and safety in numerous solid tumors [53]. It is therefore important to assess its effectiveness in treating HCC. In this study, the associations between the radiomics nomogram and several immunotherapy biomarkers were assessed. High levels of immune checkpoint genes and tumor mutation burden are generally associated with immunotherapy sensitivity [31, 33]. Decreased *HLA* expression can impair the antigen-presenting capacity of immune cells, leading to immune escape [32]. In this study, the immune checkpoint *TNFSF9*, *HLA-C*, and *TP53* mutations were upregulated in the high-score group. *TP53* mutations have been identified as a main genomic characteristic of proliferative HCC in a previous study [4]. From this perspective, the proliferative HCC prediction radiomics nomogram might non-invasively detect immune dysfunction and serve as a potential biomarker of immunotherapy benefits.

Pathomics, which could provide vast amounts of detailed information about cancer progression, is a promising tool for exploring the heterogeneity of tumor cells [22, 23]. Wang et al. revealed the relationship between radiomics and pathomics in breast cancer [17]. However, the relationship in HCC has not been explored until now. Our study found that both global patterns of the HCC tumor cell (e.g. texture of the nuclei and cytoplasm) and local anatomical structures (e.g. shape of the nuclei) from pathomics data were associated with the radiomics nomogram. Therefore, we hypothesized that morphological changes of HCC reflected by radiomics could result from the heterogeneity of tumor cells.

Our study has several limitations. First, the study’s retrospective nature and reliance on specific datasets could limit the generalizability of findings to broader populations or different clinical settings. Second, hepatitis virus infection was the leading cause of HCC in this study. The model will need to be validated with various HCC etiologies and associated prognoses. Third, this multi-omics study explored a link between radiomics, transcriptomics, and pathomics using the TCIA database. In the future, it will be necessary to incorporate additional omics data, such as proteomics or metabolomics data, to provide a more comprehensive understanding of the tumor microenvironment and its implications for HCC progression and treatment response.

In conclusion, a non-invasive prediction of proliferative HCC was achieved using a CT-based radiomics nomogram, which was associated with a pro-tumor microenvironment. The nomogram may serve as a valuable tool to predict aggressive tumor behaviors and guide personalized therapeutic strategies.

### Supplementary Information

Supplementary material 1.

## Data Availability

The datasets generated and analyzed during the current study are available by the corresponding author upon reasonable request.

## Abbreviations

AFP: α-Fetoprotein
AUC: Area under the receiver operating characteristic curve
CI: Confidence interval
CT: Computed tomography
HCC: Hepatocellular carcinoma
HR: Hazard ratio
MRI: Magnetic resonance imaging
RFS: Recurrence-free survival
OR: Odds ratio
PFS: Progression-free survival
TACE: Transarterial chemoembolization
TCIA: The cancer imaging archive
TCGA: The cancer genome atlas
