# Supplementary material for: CT-based radiomics nomogram to predict proliferative hepatocellular carcinoma and explore the tumor microenvironment
Source: J Transl Med. 2024 Sep 2;22:683. doi: 10.1186/s12967-024-05393-3 (PMC11367757; doi:10.1186/s12967-024-05393-3)
Supplement: Supplementary file 1 — Supplementary material 1. [file 12967_2024_5393_MOESM1_ESM.docx]

# Supplementary material

## Appendix S1: Survival analysis

Patients underwent clinical and radiological (CT or MRI) follow-up every 3–6 months following treatment. Early RFS was defined as the time from surgery to tumor recurrence within 24 months after treatment. In the surgery outcome cohort, patients were stratified into low-score (bottom 1/3 of predicted scores) and high-score (top 2/3 of predicted scores) groups using the radiomics nomogram. There were 105 patients who received TACE therapy for HCC and had paired clinical data and CT images in the TCIA database (TCIA-TACE-Seg). Of these patients, one patient was excluded because of incomplete CT images and the remaining 104 patients were included in the TACE outcome cohort. The PFS was defined as the time from first TACE procedure to tumor progression or death. Patients were stratified into low-score (bottom of 1/3 predicted scores) and high-score (top 2/3 of predicted scores) groups using the radiomics nomogram. The Kaplan-Meier curves were generated and a log-rank test was performed in the two outcome cohorts.

## Appendix S2: Analysis of pathomics features

The 33 patients in the bioinformatics cohort had paired Hematoxylin-eosin-stained histology images of HCC, and these data were used for subsequent pathomics analyses. Patients were predicted as nonproliferative HCC (low-score group, n = 15) or proliferative HCC (high-score group, n = 18) using the radiomics nomogram. The histology image data in SVS format were read and processed using Aperio ImageScope software (Leica). Five representative tumor regions containing the greatest number of tumor cells were selected by two experienced pathologists (H.C. and F.C.) for each slide and were exported in TIFF format (1000×1000 pixels).

To extract objective pathomics features from slides, we built a pipeline using the CellProfiler software (cellprofiler.org) as described in previous studies [1-3]. With the “UnmixColors” module, the pipeline first unmixed representative images to identify the foreground of targeted tissue. Second, the pipeline recognized the cell nuclei and cytoplasm using the “IdentifyPrimaryObjects” and “IdentifySecondObjects” modules with adaptive Otsu thresholds. Next, pathomics features reflecting the shapes, sizes, textures, pixel intensity distributions and proximity relations of the primary and secondary objects were extracted using the “MeasureObjectSizeShape,” “MeasureTexture,” “MeasureObjectIntensity” and “MeasureObjectIntensityDistribution” modules. A total of 1362 pathomics features were extracted and aggregated by mean, median, and standard deviation of the values. The categories of pathomics features are shown in Table S6. The values of pathomics features from five representative regions were averaged for subsequent analysis. We explored the radiomics nomogram-associated pathomics features using the “DESeq” package with R software. A threshold of *P* < 0.05 was set.

## Appendix S3: Formulas of the clinical model, radiomics model, and radiomics nomogram. Note. AFP = α-fetoprotein.

Clinical model score = 1/(1+exp(-logit)), where logit = 0.3559×AFP+(-0.3249)×age+(-0.3939)×albumin+(-0.0608)

Fusion* radiomics nomogram score = 1/(1+exp(-logit)), where logit = 0.8772×fusion* radscore+0.4045×AFP+(-0.3683)×age+(-0.3650)×albumin+(-0.1749)

Fusion radiomics nomogram score = 1/(1+exp(-logit)), where logit = 1.3395×fusion radscore+0.3400×AFP+(-0.4888)×age+(-0.1663)×albumin+(-0.2800)

## Table S1: CT Image filtering preprocessing methods and explanation

| Image filter (10 types) | Explanation |
| --- | --- |
| Original | No filter applied |
| Wavelet | Wavelet filtering, yields 8 decompositions per level (all possible combinations of applying either a High or a Low pass filter in each of the three dimensions. |
| LoG | Laplacian of Gaussian filter, edge enhancement filter. Emphasizes areas of gray level change, where sigma defines how coarse the emphasised texture should be. A low sigma emphasis on fine textures (change over a short distance), where a high sigma value emphasises coarse textures (gray level change over a large distance). |
| Square | Takes the square of the image intensities and linearly scales them back to the original range. Negative values in the original image will be made negative again after application of filter. |
| SquareRoot | Takes the square root of the absolute image intensities and scales them back to original range. Negative values in the original image will be made negative again after application of filter. |
| Logarithm | Takes the logarithm of the absolute intensity + 1. Values are scaled to original range and negative original values are made negative again after application of filter. |
| Exponential | Takes the the exponential, where filtered intensity is e^(absolute intensity). Values are scaled to original range and negative original values are made negative again after application of filter. |
| Gradient | Returns the gradient magnitude. |
| LBP2D | Calculates and returns a local binary pattern applied in 2D. |
| LBP3D | Calculates and returns local binary pattern maps applied in 3D using spherical harmonics. Last returned image is the corresponding kurtosis map. |

## Table S2: Selected features included in six radiomics models

| Radiomics models | Selected features |
| --- | --- |
| Plain (n = 12) | log-sigma-2-0-mm-3D_gldm_LargeDependenceLowGrayLevelEmphasis  wavelet-HLL_glcm_DifferenceVariance  wavelet-HLH_firstorder_Skewness  lbp-3D-k_ngtdm_Strength  log-sigma-2-0-mm-3D_ngtdm_Busyness  lbp-2D_firstorder_90Percentile  logarithm_glrlm_RunLengthNonUniformity  log-sigma-1-0-mm-3D_firstorder_Skewness  wavelet-HHH_glszm_LargeAreaLowGrayLevelEmphasis  exponential_firstorder_Skewness |
| Arterial (n = 10) | log-sigma-2-0-mm-3D_gldm_LargeDependenceLowGrayLevelEmphasis  wavelet-HLL_glcm_DifferenceVariance  wavelet-HLH_firstorder_Skewness  lbp-3D-k_ngtdm_Strength  log-sigma-2-0-mm-3D_ngtdm_Busyness  lbp-2D_firstorder_90Percentile  logarithm_glrlm_RunLengthNonUniformity  log-sigma-1-0-mm-3D_firstorder_Skewness  wavelet-HHH_glszm_LargeAreaLowGrayLevelEmphasis  exponential_firstorder_Skewness |
| Venous (n = 8) | log-sigma-2-0-mm-3D_gldm_LargeDependenceLowGrayLevelEmphasis  wavelet-HLL_glcm_DifferenceVariance  wavelet-HLH_firstorder_Skewness  lbp-3D-k_ngtdm_Strength  log-sigma-2-0-mm-3D_ngtdm_Busyness  lbp-2D_firstorder_90Percentile  logarithm_glrlm_RunLengthNonUniformity  log-sigma-1-0-mm-3D_firstorder_Skewness |
| Delayed (n = 11) | log-sigma-2-0-mm-3D_gldm_LargeDependenceLowGrayLevelEmphasis  wavelet-HLL_glcm_DifferenceVariance  wavelet-HLH_firstorder_Skewness  lbp-3D-k_ngtdm_Strength  log-sigma-2-0-mm-3D_ngtdm_Busyness  lbp-2D_firstorder_90Percentile  logarithm_glrlm_RunLengthNonUniformity  log-sigma-1-0-mm-3D_firstorder_Skewness  wavelet-HHH_glszm_LargeAreaLowGrayLevelEmphasis  exponential_firstorder_Skewness  log-sigma-2-0-mm-3D_gldm_LargeDependenceLowGrayLevelEmphasis |
| Fusion* (n = 13) | log-sigma-2-0-mm-3D_gldm_LargeDependenceLowGrayLevelEmphasis_A  wavelet-LLH_glcm_MaximumProbability_V  wavelet-HLL_glcm_DifferenceVariance_A  log-sigma-2-0-mm-3D_glcm_ClusterProminence_V  wavelet-HLH_firstorder_Skewness_A  log-sigma-3-0-mm-3D_firstorder_Skewness_V  log-sigma-4-0-mm-3D_firstorder_Range_V  squareroot_firstorder_Maximum_V  lbp-3D-k_ngtdm_Strength_A  log-sigma-2-0-mm-3D_ngtdm_Busyness_A  lbp-2D_firstorder_90Percentile_A  log-sigma-1-0-mm-3D_firstorder_Skewness_A  wavelet-HHL_firstorder_Kurtosis_V |
| Fusion (n = 25) | log-sigma-2-0-mm-3D_gldm_LargeDependenceLowGrayLevelEmphasis_A  log-sigma-5-0-mm-3D_firstorder_Minimum_D  wavelet-LLH_glcm_MaximumProbability_V  log-sigma-1-0-mm-3D_gldm_LowGrayLevelEmphasis_P  wavelet-HLL_glcm_DifferenceVariance_A  log-sigma-2-0-mm-3D_glcm_ClusterProminence_V  square_firstorder_Skewness_P  original_firstorder_Skewness_P  wavelet-HLH_firstorder_Skewness_A  log-sigma-4-0-mm-3D_ngtdm_Strength_D  log-sigma-3-0-mm-3D_firstorder_Skewness_V  squareroot_firstorder_Maximum_V  lbp-3D-m2_firstorder_Kurtosis_P  exponential_glszm_SizeZoneNonUniformity_D  log-sigma-2-0-mm-3D_ngtdm_Busyness_A  lbp-3D-k_glcm_Imc1_P  logarithm_glrlm_RunLengthNonUniformity_P  wavelet-LHL_firstorder_Skewness_P  lbp-3D-k_glrlm_RunLengthNonUniformityNormalized_P  lbp-2D_firstorder_90Percentile_A  lbp-2D_firstorder_InterquartileRange_P  logarithm_glcm_ClusterShade_D  wavelet-HHL_firstorder_Kurtosis_V  log-sigma-3-0-mm-3D_glcm_Imc1_P  wavelet-LHH_firstorder_Skewness_P |

Note. P = plain, A = arterial, V = portal venous, D = delayed.

Fusion* radiomics model is based on arterial and portal venous phase images, and fusion radiomics model is based on all four phases of images.

## Table S3. Pathomics feature categories extracted by CellProfiler software

| Feature Category | CellProfiler Module | Feature Examples |
| --- | --- | --- |
| Cytoplasm Size and Shape,  Nucleus Size and Shape | MeasureObjectSizeShape | Area,  perimeter,  Zernike shape features |
| Cytoplasm Texture,  Nucleus Texture | MeasureTexture | Entropy,  Contrast,  Variance |
| Pixel Intensity of the Cytoplasm,  Pixel Intensity of the Nuclei | MeasureObjectIntensity | Mean intensity,  lower quartile intensity,  upper quartile intensity |
| Radial Distribution of Pixel Intensity of the Cytoplasm,  Radial Distribution of Pixel Intensity of the Nuclei | MeasureObjectIntensityDistribution | FracAtD,  MeanFrac,  RadialCV |

## Table S4. Representative pathomics features identified by radiomics nomogram

| Pathomics features | LogFC | *P* Value |
| --- | --- | --- |
| Median_Nuclei_Texture_SumEntropy_Hematoxylin_3_02_256 | -0.20 | 0.03 |
| Mean_Cytoplasm_Texture_DifferenceEntropy_Hematoxylin_3_03_256 | -0.22 | 0.04 |
| Median_Nuclei_AreaShape_BoundingBoxMaximum_X | 4.12 | 0.04 |
| Mean_Nuclei_RadialDistribution_MeanFrac_Hematoxylin_1of4 | -0.04 | 0.04 |
| Median_Cytoplasm_Texture_SumVariance_Hematoxylin_3_02_256 | -653.91 | 0.04 |
| Median_Nuclei_Location_Center_X | 4.05 | 0.04 |
| Mean_Cytoplasm_Texture_Contrast_Hematoxylin_3_03_256 | -495.37 | 0.04 |
| Median_Nuclei_Location_CenterMassIntensity_X_Hematoxylin | 4.03 | 0.04 |
| StDev_Cytoplasm_Intensity_IntegratedIntensityEdge_Hematoxylin | 2.13 | 0.04 |
| Median_Nuclei_Intensity_MeanIntensityEdge_Hematoxylin | 0.08 | 0.04 |
| Median_Cytoplasm_Texture_Variance_Hematoxylin_3_03_256 | -266.22 | 0.04 |
| Mean_Cytoplasm_Texture_Entropy_Hematoxylin_3_02_256 | -0.12 | 0.04 |
| Median_Nuclei_Location_MaxIntensity_X_Hematoxylin | 3.84 | 0.04 |
| Mean_Nuclei_Texture_DifferenceEntropy_Hematoxylin_3_00_256 | -0.23 | 0.045 |

Note. The logFC and *p* values are calculated using the“DESeq” package. logFC = log2 (fold change).


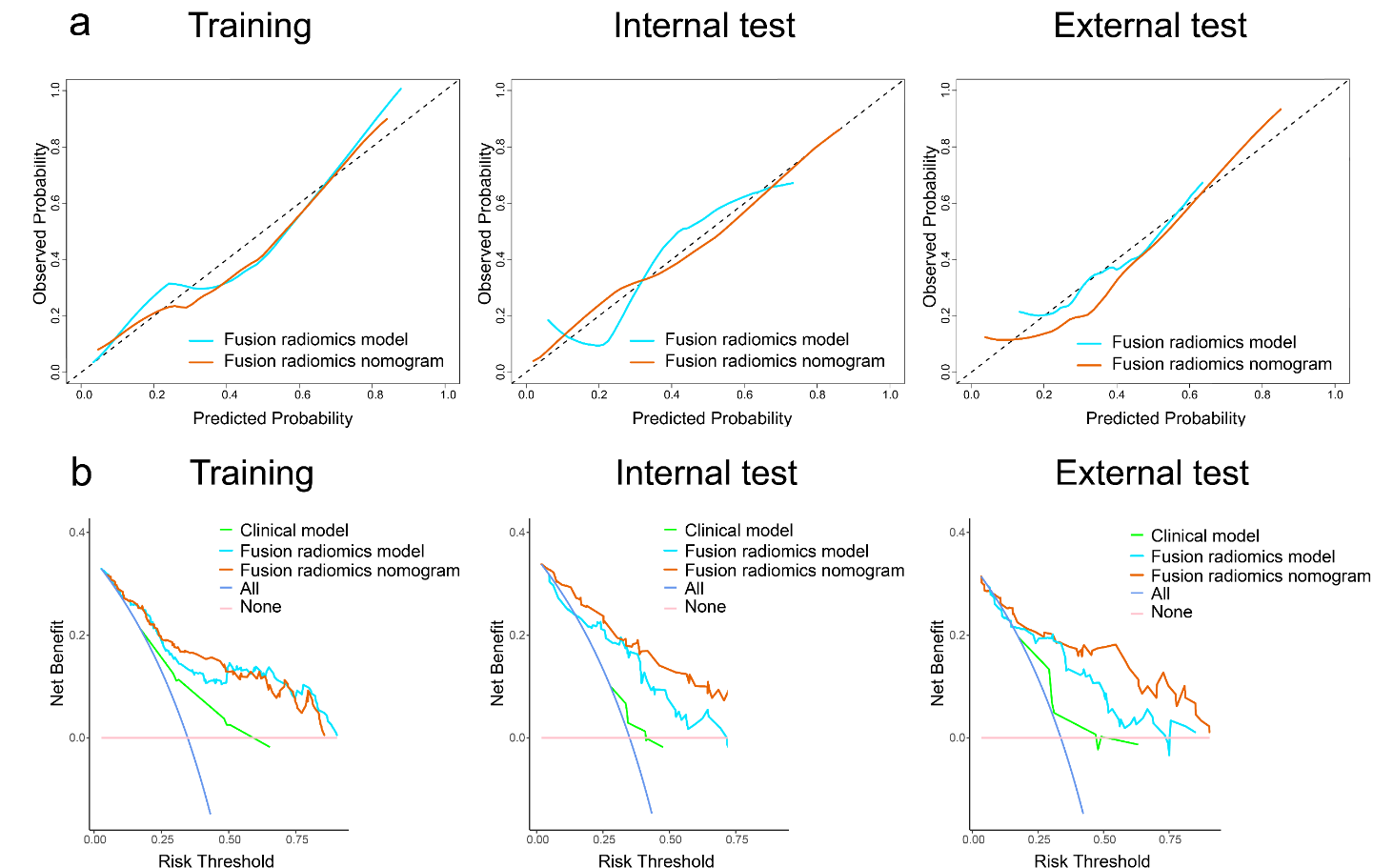


## Fig. S1 (a) The calibration curves and (b) decision curves of fusion radiomics model and fusion radiomics nomogram in the train, internal test, and external cohorts.


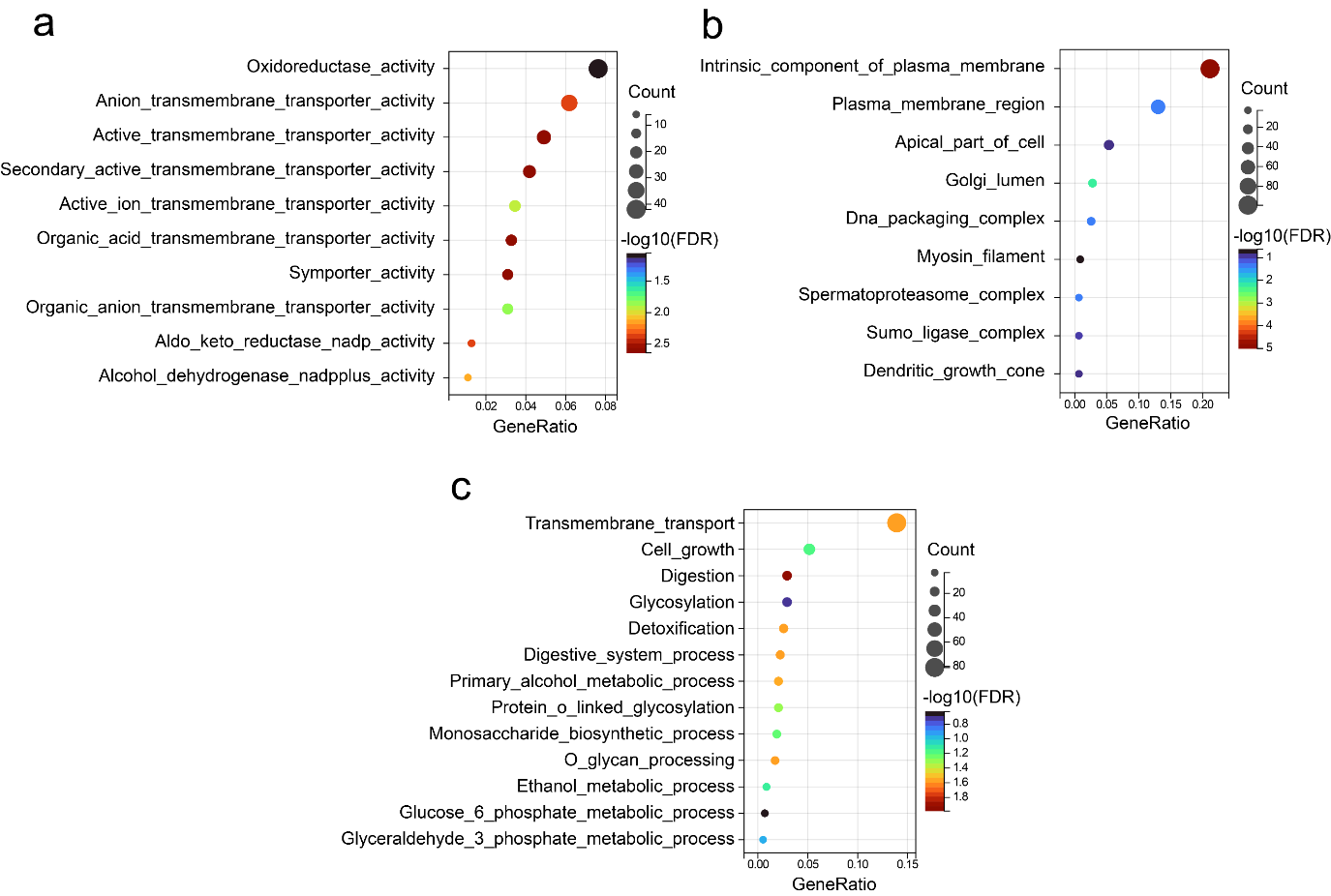


## Fig. S2 Bubble plots show results of the functional enrichment analysis using Gene Ontology, including (a) molecular function, (b) cellular component, and (c) biological process. GeneRatio means the ratio of genes in this pathway to all genes. Gene Ontology analysis was performed by “clusterProfiler” package with thresholds of *P* < 0.05 and false discovery rate < 0.25.


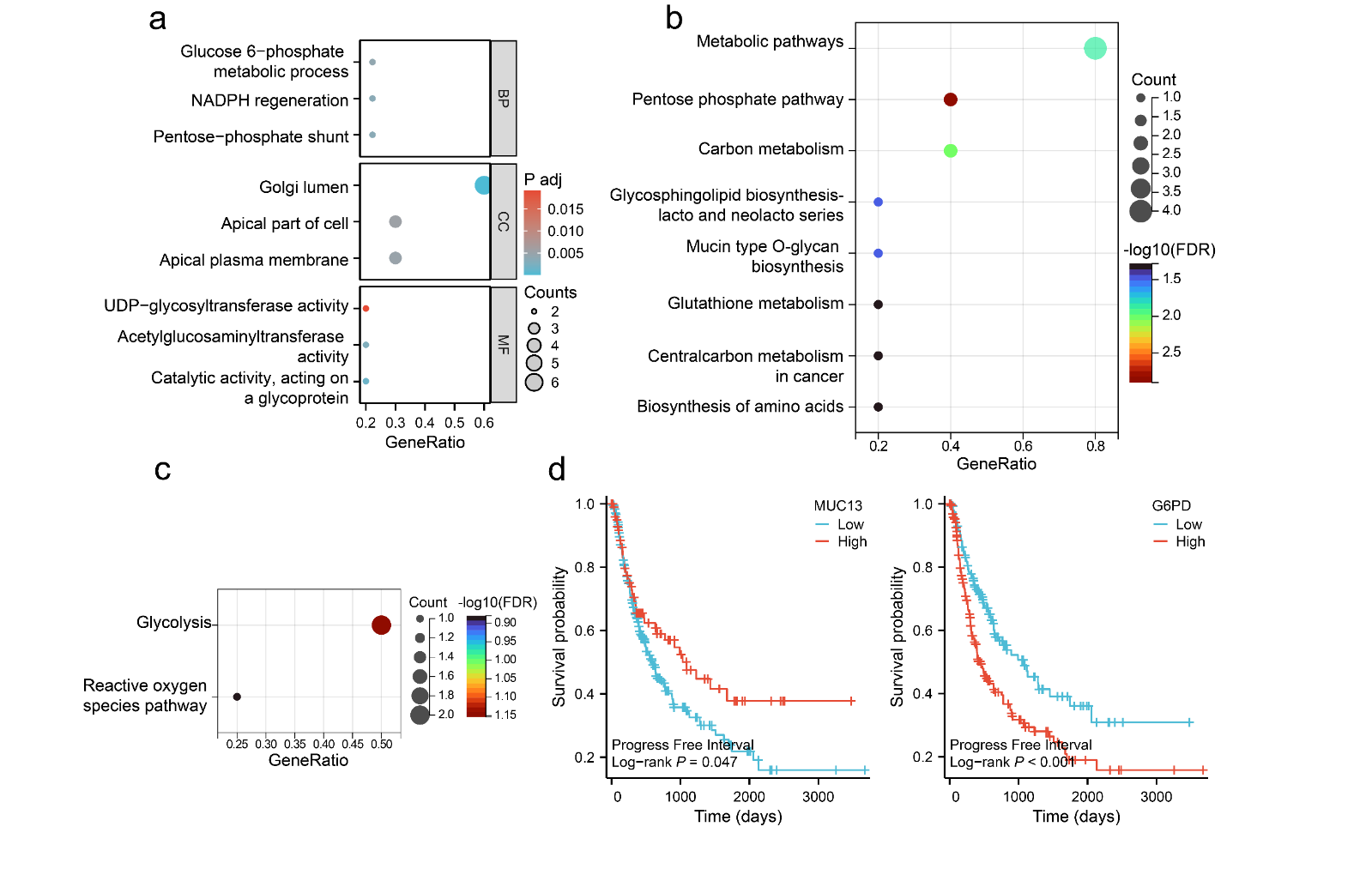


## Fig. S3 Functional enrichment analysis of the hub genes using (a) Gene Ontology, (b) Kyoto Encyclopedia of Genes and Genomes pathways, and (c) hallmark gene sets. GeneRatio means the ratio of genes in this pathway to all genes. The functional enrichment analyses were performed by “clusterProfiler” package with thresholds of *P* < 0.05 and false discovery rate < 0.25. (d) Two representative hub genes are correlated with progression-free interval.


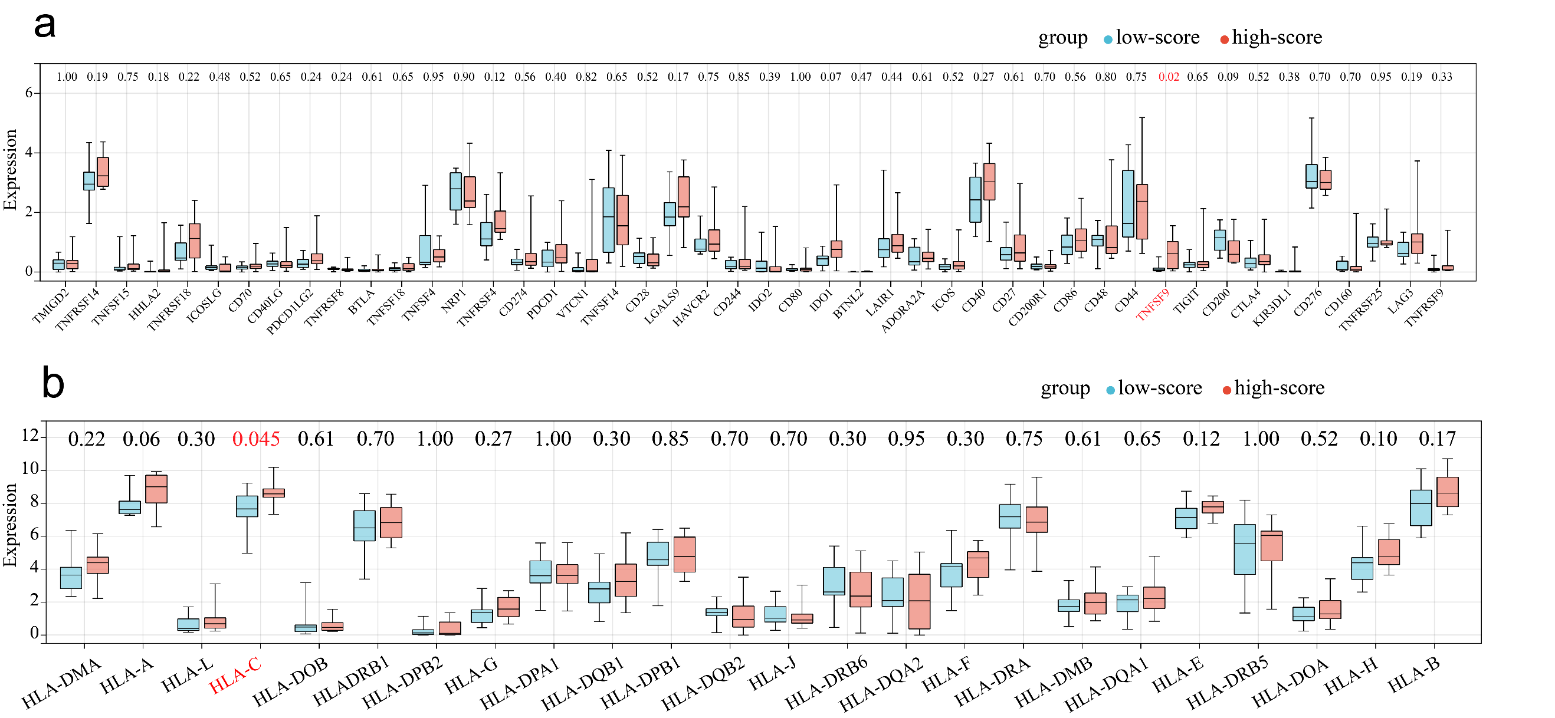


## Fig. S4 Analysis of (a) immune checkpoints and (b) HLA members between low-score and high-score groups.

**References:**

1 Carpenter AE, Jones TR, Lamprecht MR et al (2006) CellProfiler: image analysis software for identifying and quantifying cell phenotypes. Genome Biol 7:R100

2 Yu KH, Zhang C, Berry GJ et al (2016) Predicting non-small cell lung cancer prognosis by fully automated microscopic pathology image features. Nat Commun 7:12474

3 Chen S, Zhang N, Jiang L et al (2021) Clinical use of a machine learning histopathological image signature in diagnosis and survival prediction of clear cell renal cell carcinoma. Int J Cancer 148:780-790
